# An automated positive selection screen in yeast provides support for boron-containing compounds as inhibitors of SARS-CoV-2 main protease

**DOI:** 10.1128/spectrum.01249-24

**Published:** 2024-08-20

**Authors:** Sunniva Sigurdardóttir, Suélen Fernandes Silva, Ievgeniia Tiukova, Hanna Alalam, Ross D. King, Morten Grøtli, Leif A. Eriksson, Per Sunnerhagen

**Affiliations:** 1 Department of Chemistry and Molecular Biology, University of Gothenburg, Göteborg, Sweden; 2 Chemistry Institute, São Paulo State University, Araraquara, Brazil; 3 Department of Biology and Biological Engineering, Chalmers, Göteborg, Sweden; University of Manitoba, Winnipeg, Manitoba, Canada

**Keywords:** COVID-19, drug repurposing, *Saccharomyces cerevisiae*

## Abstract

The severe acute respiratory syndrome coronavirus 2 (SARS-CoV-2) virus continues to cause severe disease and deaths in many parts of the world, despite massive vaccination efforts. Antiviral drugs to curb an ongoing infection remain a priority. The virus-encoded 3C-like main protease (MPro; nsp5) is seen as a promising target. Here, with a positive selection genetic system engineered in *Saccharomyces cerevisiae* using cleavage and release of MazF toxin as an indicator, we screened in a robotized setup small molecule libraries comprising ~2,500 compounds for MPro inhibitors. We detected eight compounds as effective against MPro expressed in yeast, five of which are characterized proteasome inhibitors. Molecular docking indicates that most of these bind covalently to the MPro catalytically active cysteine. Compounds were confirmed as MPro inhibitors in an *in vitro* enzymatic assay. Among those were three previously only predicted *in silico*; the boron-containing proteasome inhibitors bortezomib, delanzomib, and ixazomib. Importantly, we establish reaction conditions *in vitro* preserving the MPro-inhibitory activity of the boron-containing drugs. These differ from the standard conditions, which may explain why boron compounds have gone undetected in screens based on enzymatic *in vitro* assays. Our screening system is robust and can find inhibitors of a specific protease that are biostable, able to penetrate a cell membrane, and are not generally toxic. As a cellular assay, it can detect inhibitors that fail in a screen based on an *in vitro* enzymatic assay using standardized conditions, and now give support for boron compounds as MPro inhibitors. This method can also be adapted for other viral proteases.

**IMPORTANCE:**

The coronavirus disease 2019 (COVID-19) pandemic triggered the realization that we need flexible approaches to find treatments for emerging viral threats. We implemented a genetically engineered platform in yeast to detect inhibitors of the virus’s main protease (MPro), a promising target to curb severe acute respiratory syndrome coronavirus 2 (SARS-CoV-2) infections. Screening molecule libraries, we identified candidate inhibitors and verified them in a biochemical assay. Moreover, the system detected boron-containing molecules as MPro inhibitors. Those were previously predicted computationally but never shown effective in a biochemical assay. Here, we demonstrate that they require a non-standard reaction buffer to function as MPro inhibitors. Hence, our cell-based method detects protease inhibitors missed by other approaches and provides support for the boron-containing molecules. We have thus demonstrated that our platform can screen large numbers of chemicals to find potential inhibitors of a viral protease. Importantly, the platform can be modified to detect protease targets from other emerging viruses.

## INTRODUCTION

Since the start of the coronavirus disease 2019 (COVID-19) pandemic, numerous attempts to find antiviral agents able to interfere with a severe acute respiratory syndrome coronavirus 2 (SARS-CoV-2) infection have been made. Drug repurposing to find small molecules hitting virus-encoded protein targets has been a leading strategy due to a much faster route to market. Proteases stand out as promising drug targets, being part of a highly druggable protein class. The SARS-CoV-2 main protease (MPro; nsp5) is required for viral replication and has attracted interest as a target for drugs aiming at the early phase of viral infection. Its biological role is to cleave the viral polyprotein into functional protein components. For MPro as a target, many attempts were initially based on using *in silico* screens as the first step (1–6). To the extent that experimental validation of those *in silico* predictions was accomplished, this has been performed with *in vitro* enzymatic activity assays (7, 8), cellular test systems (9, 10), alternatively viral infection models in animals (11, 12), or with cultured animal cell lines (10).

Cellular assay systems capture the ability of compounds to pass the plasma membrane, remain stable in the intracellular environment, and locate the correct compartment for interaction with the target. For instance, in the intracellular “FlipGFP” reporter system, MPro cleavage allows the maturation of the chromophore, and MPro inhibition can be scored as reduced green fluorescent protein (GFP) fluorescence (13, 14). A target-based screening system in cells using positive selection gives several advantages. First, only compounds that are both able to pass the cell membrane and stable enough to remain active in an intracellular environment will score in the assay. Second, using a positive selection mode filters out compounds with only general toxicity, which in a negative growth selection mode often outnumber the true hits. Third, artifacts from inappropriate *in vitro* buffer systems are avoided. Finally, effects on the target can be discriminated from off-target effects. In the case of viruses, both host cell and virus-encoded proteases can be involved in the progression of a viral infection, and a protease inhibitor can interact with more than one of these. In that respect, genetically modified yeast (*Saccharomyces cerevisiae*) is a suitable system in which to construct cellular assays for inhibition of SARS-CoV-2 protein targets (15, 16).

Here, we have screened three focused compound libraries using a genetic system in *S. cerevisiae* that we recently constructed to detect MPro inhibitors using positive selection (17). In this system, the *E. coli* MazEF toxin fusion is expressed in yeast as a detector of protease activity. MazF is an endoribonuclease that cleaves mRNA at the sequence ACA (18). In *E. coli*, its activity is quenched by the antitoxin MazE (19). A MazEF translational fusion was genetically engineered by joining MazE to the MazF N-terminus through a peptide linker with an MPro consensus cleavage site inserted. Upon expression of MPro and MazEF in yeast, MPro cleaves the toxin/antitoxin MazEF fusion, releasing active MazF resulting in decreased cell proliferation. The presence of an MPro inhibitor then prevents MazEF cleavage and rescues cell growth. This system thus presents a positive selection for MPro inhibitors, discriminating the many compounds with non-specific cytotoxic effects from the less numerous actual inhibitors of the target enzyme (17).

Using this genetic system in yeast, we screened approximately 2,500 compounds, representing approved drugs, drug-like molecules, and molecules with available human test data. We identified a small number conferring increased growth in the reporter strain, as expected from MPro inhibitors. The positive selection mode further allowed the filtering out of molecules with cytotoxic effects. Secondary testing in yeast cells not expressing MPro ensured that the response to the compounds was indeed dependent on the presence of MPro. Using an *in vitro* enzymatic inhibition assay and *in silico* docking to the catalytic site of the target protein, we verified that the identified candidate MPro inhibitors fit the binding site and are likely to interact directly with the target protein. The majority of MPro inhibitor candidates identified were previously characterized as proteasome inhibitors, in line with published data (7, 20–30). However, we also detected a group of boron-containing proteasome-inhibitor drugs; bortezomib, delanzomib, and ixazomib, that had not previously been recognized in functional assays. For these novel MPro inhibitors, we had to adapt the buffer conditions for the enzymatic assay to demonstrate their capacity to inhibit MPro *in vitro*.

Thus, the genetic screening system in yeast that we previously constructed has been validated as a reliable tool for high-throughput identification of protease inhibitors that function in living cells. We also demonstrate that a cellular screening assay, in addition to selecting only the molecules capable of entering cells, can find hits that were missed in screens based on *in vitro* enzymatic assays because of inappropriate reaction buffer conditions. The boron-containing molecules identified this way are in clinical use as anticancer drugs. The complex chemistry of boron makes such compounds versatile with potential for drug development. This screening platform can also easily be adapted to proteases from other viral or infectious diseases, provided the peptide sequence of the cleavage site is known.

## RESULTS

### Screening-focused libraries using positive selection in yeast

To screen for MPro inhibitors, an *S. cerevisiae* strain expressing MPro and a positive selection plasmid reporter was used as previously described (17). The reporter expresses the MazEF chimera with an MPro consensus cleavage site inserted in the linker between the MazF toxin and MazE antitoxin. The strain also carries deletions of the *SNQ2*, *PDR1*, and *PDR3* genes encoding one ABC membrane transporter and two transcription factors regulating the expression of several ABC transporters, respectively (17). This sensitizes the cell to external small molecules without significant negative effects on strain hardiness (31). To allow for monitoring growth by fluorescence, the strain constitutively expresses mCherry from a chromosomal locus (17, 32), as a marker for cell abundance. The strain was screened with a total of 2,478 unique compounds from three focused chemical libraries with partial overlap (FDA-approved, protease inhibitors and the MMV Covid Box; see Materials and Methods). Libraries were screened in a robotic platform for streamlined high-throughput screening (17, 32).

In a pilot experiment, the strain was screened with 88 compounds at 30 µM using both fluorescence and absorbance readout. In the fluorescence channel, we could detect seven compounds with a significant increase in yield over solvent-only control, while in the absorbance channel, none were discernible over noise (Fig. S1). Therefore, subsequent batches were run using the fluorescence channel only, which reduced measuring time and allowed more time in optimal growth conditions. The overall median relative standard deviation of yield was 2.3%, and the mean relative standard deviation was 7.4%, indicating little variation between technical replicates (Table S1). To assess biological and batch-to-batch variation, we compared results for compounds that were represented in more than one library. Bortezomib and delanzomib were represented in all three libraries and were confidently identified as hits in all (Table S1).

Simeprevir was also represented in all three libraries but only scored as a marginal hit in one (highest yield ratio 1.09 ± 0.02). This indicates some batch-to-batch variation, and we judged that to confidently identify a hit, a yield increase of ≥10% is needed in our assay. However, simeprevir did inhibit MPro *in vitro* (Table 1), and so this may indicate inefficient uptake in cells. Two compounds that we previously tested as negative in the assay (17) were again tested herein. In agreement with the earlier results, boceprevir (included in all three libraries) had only slightly cytotoxic effects, and glycyrrhizic acid (represented in one library) had no effect (Table S1).

**TABLE 1:** Candidate inhibitors^*a*^

	Yeast assay		Enzymatic assay			Molecular docking		
Compound	FC of yield at 30 µM^*b*^	FC of yield at 100 µM^*b*^	IC_50_ (µM)	IC_20_ (µM)	R_2_	cDock affinity (kcal/mol)^*e*^	MMGBSA (kcal/mol)^*f*^	XP docking score (kcal/mol)^*g*^
Antiviral								
GC376	2.59 ± 0.07***^c^	3.67 ± 0.34***^c^	0.036	0.015	0.997	−9.92	−67.8	−9.36
Simeprevir	0.97 ± 0.02	1.13 ± 0.04*	84.49	27.29	0.98	ND	ND	−2.14
Proteasome inhibitors								
Bortezomib	1.28 ± 0.03***	1.27 ± 0.02***	277^*d*^	39.34	0.968	−7.55	−41.5	−10.05
Delanzomib	1.28 ± 0.02***	1.32 ± 0.01***	217.9^*d*^	37.64	0.973	−8.94	−41.3	−9.43
Ixazomib	1.25 ± 0.01***	1.22 ± 0.05***	121.5^*d*^	24.43	0.989	−9.18	−33.1	−7.30
MG132	0.96 ± 0.14	1.46 ± 0.07***	41.45	7.38	0.991	−8.59	−33.1	−6.88
Calpeptin	1.44 ± 0.01***	1.89 ± 0.02***	9.36	3.14	0.999	−8.29	−22.7	−8.43
Oprozomib	1.06 ± 0.02*	0.23 ± 0.01***	310.5^*d*^	114.2^*d*^	0.943	−7.91	−43.4	−7.87
Carfilzomib	1.14 ± 0.02***	0.23 ± 0.00***	ND	ND	0.72	−8.61	−55.6	−9.53
Other								
Z-VAD-FMK	1.1 ± 0.01***	1.41 ± 0.02***	0.052	0.034	0.999	−7.70	−53.6	−9.58

^*a*^ Yeast assay: GC376 12 replicates, others 4 replicates; enzymatic assay: triplicates; * adjusted *P* < 0.01; *** adjusted *P* < 0.001; ND: no inhibition detected (enzymatic assay)/no relevant pose predicted (molecular docking).

^*b*^ Fold change (FC) in yield is calculated as [yield(sample)/yield(DMSO)].

^*c*^ Data from Alalam et al. (17).

^*d*^ IC values extrapolated from available data.

^*e*^ cDock affinity corresponds to the score obtained in the covalent docking.

^*f*^ MMGBSA (dGbind) corresponds to the free binding free energy values before covalent bond formation.

^*g*^ XP (extra precision) docking score corresponds to the score obtained in the ligand docking.

**Fig 1:**
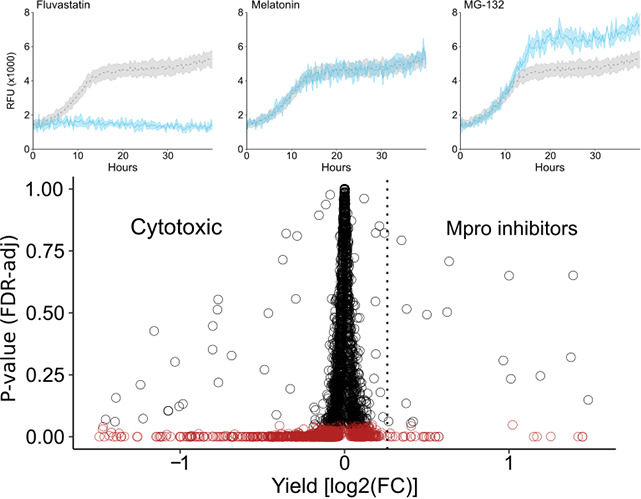
Overview of results from the primary screen in yeast. Example curves from the primary screen on top (average ± SD) in light blue with solvent-only (DMSO) as a reference (gray). Cytotoxic (Fluvastatin), no effect (Melatonin), MPro inhibitor (MG-132). Below: scatterplot of the primary screen—log_2_ of yield fold change against adjusted *P*-value (FDR corrected). The dashed vertical line indicates a 20% increase in yield, red points have *P*-value < 0.05.

**Fig 2:**
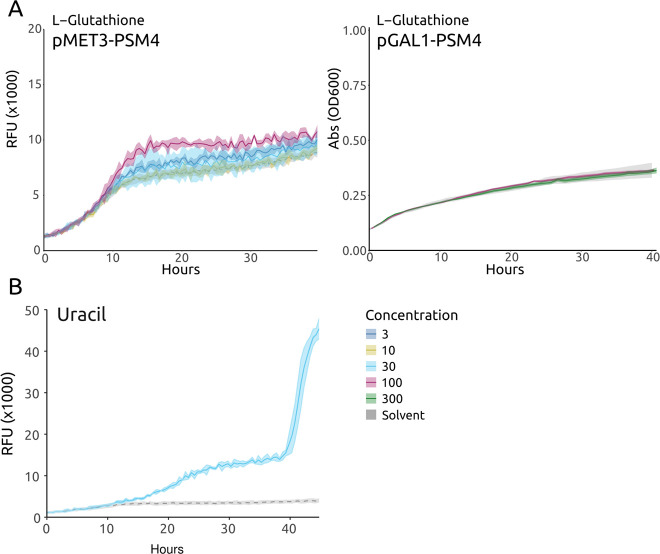
Growth curves of the yeast reporter strain expressing MPro and the toxin chimera together with compounds expected to target the toxin promoter or selection marker. (**A**) In the presence of L-glutathione, cell proliferation increases in a dose-response manner when the toxin is regulated by the *MET3* promoter (left), but when using the *GAL1* promoter (right), there is no change in growth compared to the control condition (solvent only, gray curve). (**B**) Cell proliferation increases in the presence of uracil with a huge increase toward the end of the experiment, suggesting a loss of the toxin-expressing plasmid with a uracil-selectable marker.

The vast majority of compounds had no impact on growth yield compared to the control condition (solvent only) and more samples were concluded to have cytotoxic effects than those promoting growth, as seen in the scatterplot, with a negative skew in the tail distribution (Fig. 1). Specifically, 84 compounds had at least 10% increase in yield, while 20 compounds had ≥20% yield increase (FDR-adjusted *P*-value < 0.05). On the other hand, 401 compounds decreased yield by at least 10%, indicating cytotoxic effects (FDR-adjusted *P* < 0.05; Fig. 1), These results illustrate the benefits of a positive selection system. Out of the 20 compounds with strong effect, four could be discarded as compounds down-regulating the *MET3* promoter driving toxin fusion expression (methionine and other sulfur-containing amino acids that can be metabolized to methionine) (Fig. 2A). One compound (uracil) was interfering with selection for the *URA3* marker on the plasmid expressing the toxin fusion (Fig. 2B).

**Fig 3:**
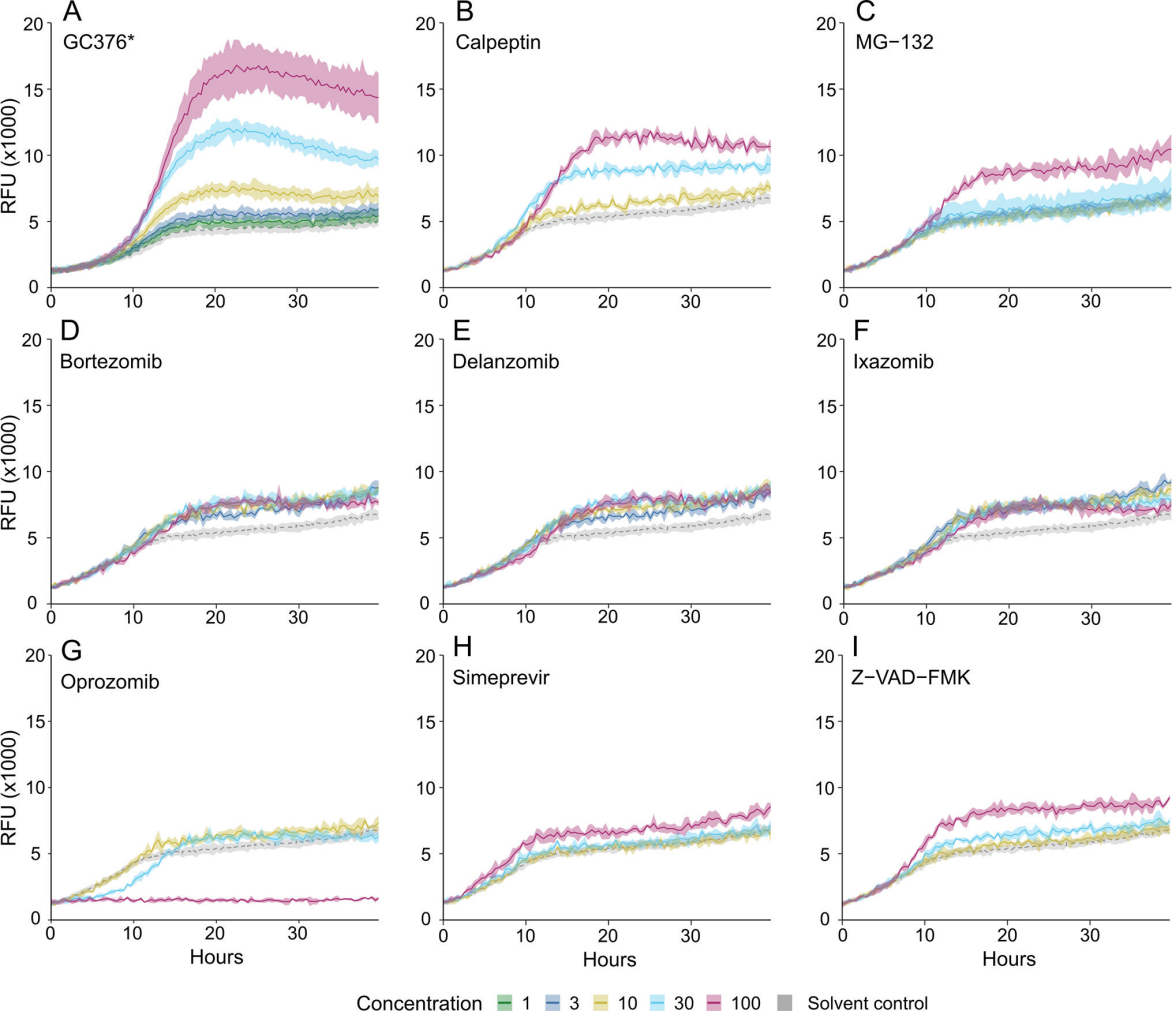
Titration curves of selected compounds with an increase in growth in the yeast reporter strain expressing MPro and the toxin chimera. The gray curve shows growth for the control condition containing solvent only. (**A**) Positive control compound GC376 was added as a reference [data from Alalam et al. (17)], (**B–I**) candidate Mpro inhibitors.

**Fig 4:**
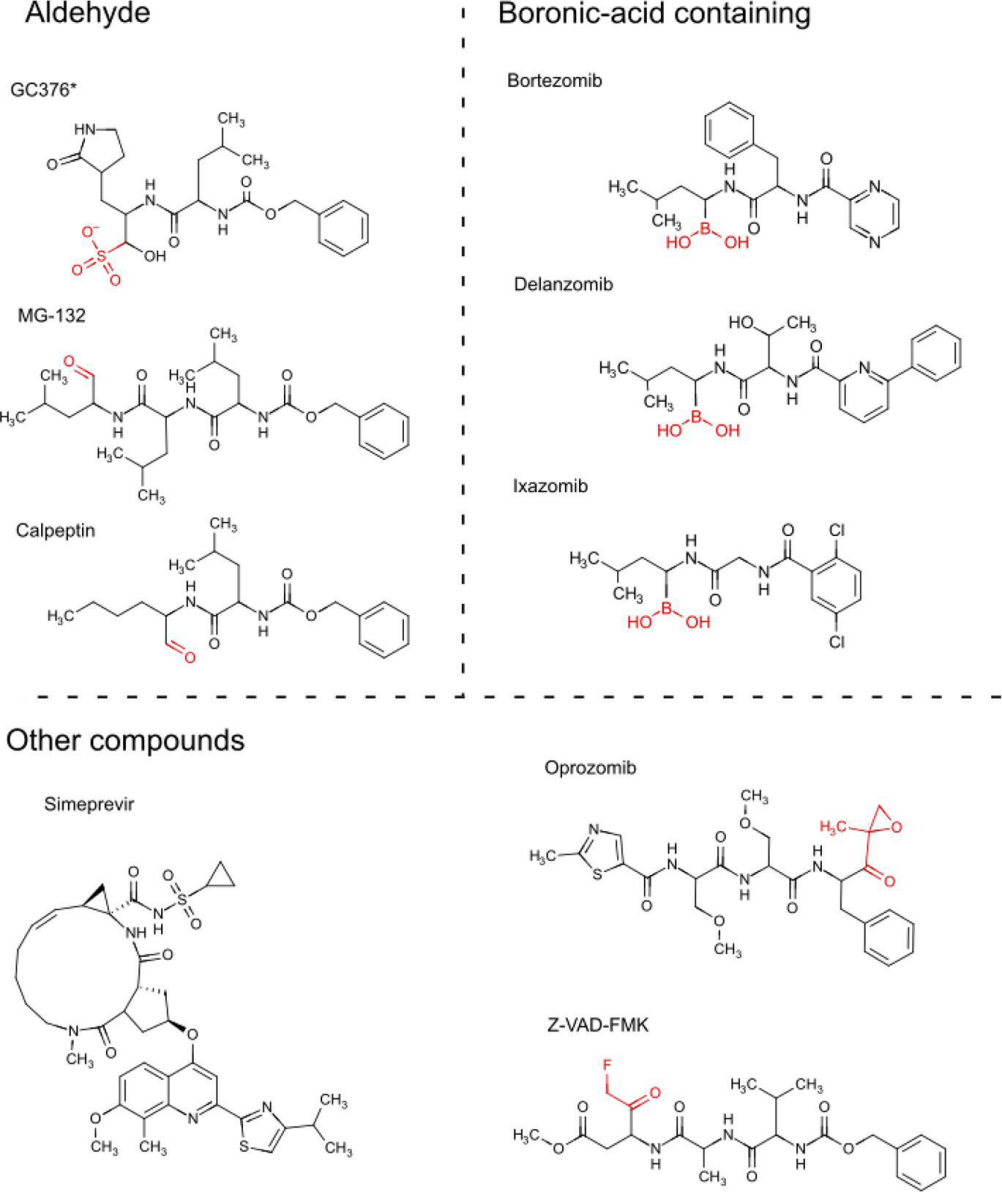
Molecule structures of candidate MPro inhibitors grouped according to functional groups/active moieties. Reactive groups are shown in red. GC376 is marked with a star to indicate that it was not represented in the chemical libraries investigated in this work. GC376 is a prodrug that upon intracellular activation loses its bisulfite group and is converted to an aldehyde.

To verify candidate hits, phenotypic assays were done in quadruplicates with compound concentrations ranging from 1 to 100 µM. Most compounds showed a graded dose response, although some compounds were cytotoxic at high concentrations (Fig. 3; Fig. S2), leaving nine compounds considered the most promising (Table 1). Of these remaining compounds, seven were known proteasome inhibitors: bortezomib, delanzomib, MG-132, ixazomib, oprozomib, carfilzomib, and calpeptin (Table 1). Common to the proteasome inhibitors is that they contain reactive functional groups/moieties, specifically boronic acid, aldehyde, or fluoromethyl ketone. These reactive groups can form covalent bonds to many nucleophiles; these proteasome inhibitors have been designed to target side chains of cysteine or threonine (33) (Fig. 4).

**Fig 5:**
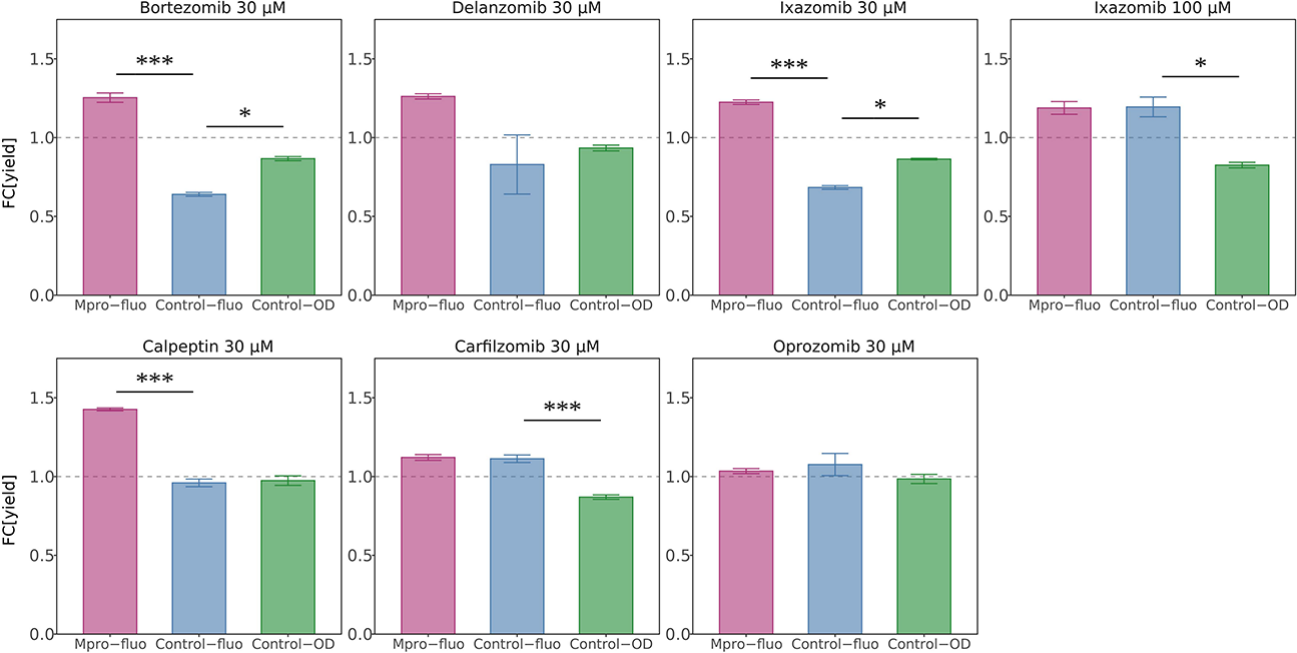
Yield ratios of compounds at 30 µM compared to solvent only in different strains. The dashed gray line indicates no difference to the control condition. Pink: strain expressing MPro and toxin chimera with fluorescence readout. Blue: control strain without MPro or toxin chimera with fluorescence readout. Green: control strain without MPro or toxin chimera with absorbance readout. Calpeptin is an example of a true hit, where only the pink bar has an increase in relative yield (above the dashed line). Carfilzomib has an increase in yield in both strains with fluorescent readout (pink and blue) while there is no increase for the control strain in absorbance readout (green). This suggests that MPro is not the main target and that the fluorescence increase is due to off-target effects. The same can be seen for Ixazomib at 100 µM, but not at 30 µM which could indicate that there are off-target or cytotoxic effects at high concentrations.

To verify that the increase in the fluorescence channel for hits in the screen was indeed MPro-dependent, they were also tested in the negative control yeast strain lacking MPro and MazEF. Most compounds had no effect on the control strain, as expected from *bona fide* Mpro inhibitors. However, for carfilzomib, ixazomib, and oprozomib, the fluorescence signal also increased in the negative control strain (Fig. 5), indicating MPro-independent activity. Importantly, there was no increase in the absorbance channel in the reporter strain (Fig. 5), meaning that proliferation was not augmented, as would have been expected from MPro inhibition. Rather, the amount of mCherry protein per cell was increasing (Fig. 5). For the epoxyketones carfilzomib and oprozomib, the fluorescence signal increased at 10 µM but was depressed at 100 µM, indicating cytotoxicity (Fig. 3; Fig. S2). For ixazomib, the fluorescent signal increase in the control strain was only seen at 100 µM but not at 10 or 30 µM (Fig. 5), which could indicate off-target effects at high concentrations.

In summary, for the three above-mentioned proteasome inhibitors—carfilzomib, ixazomib, and oprozomib—other factors than MPro inhibition influence the fluorescence readout signal and complicate the interpretation.

### Testing by *in vitro* enzymatic assay

**Fig 6:**
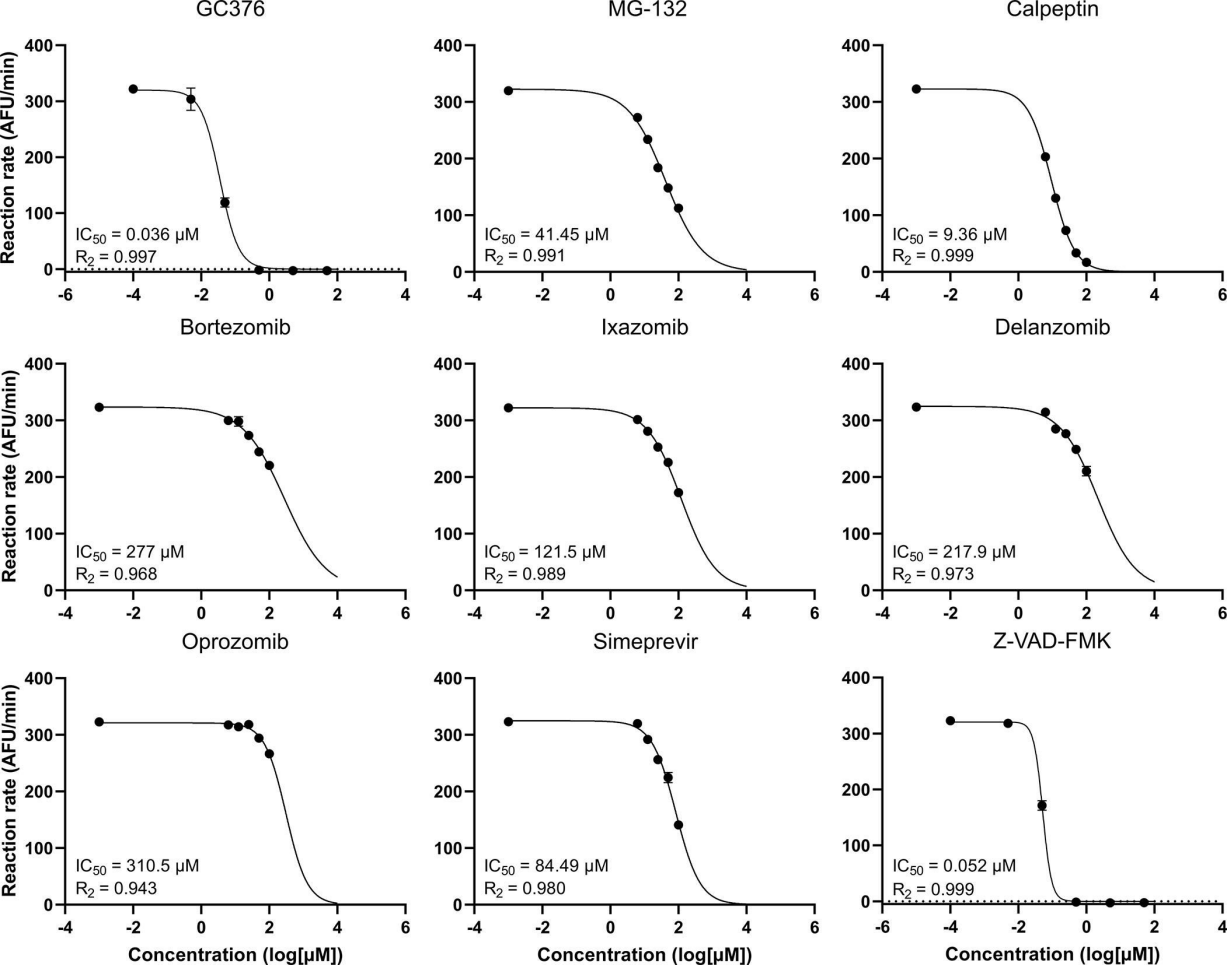
Dose-response curves of selected compounds in an enzymatic assay with MPro and the fluorogenic substrate AMC. GC376 was used as a positive control and was not represented in the chemical libraries.

The drug candidates were validated in a secondary *in vitro* assay (Fig. 6). Purified 3CL Protease (MPro) was obtained from BPS Bioscience (BPS Bioscience, San Diego, CA, USA) and a fluorophore AMC substrate was obtained from Biosynth (34). Conditions of the assay were optimized using Baker et al. (35) as a starting point, where 45 nM of the protease and 30 µM of the fluorogenic substrate were found to be suitable for our assay with a standard assay buffer (Fig. S3). All compounds were tested initially in triplicates at 50 µM. Under the standard assay conditions, we did not observe inhibition of the boron-containing compounds (bortezomib and ixazomib, Fig. S4). We speculated that the reason could be due to the redox environment *in vitro*, in light of the special reactivity of the boronic acid common to these drugs, and given that they are predicted to form a covalent bond with the target protein. Therefore, we tried changing the concentration of the reductant dithiothreitol (DTT) in the reaction buffer between 0 and 4 mM for those compounds. At 0.5 mM DTT and in the absence of a reductant, we did observe a moderate inhibitory activity of the boron-containing compounds, while inhibition of the positive control compound did not change significantly (Fig. S5A and B). Changing the conditions to pH 7.4 also showed a small increase in separation (Fig. S5C and D). Dose-response curves of all candidate hits were therefore performed without reductant and at physiological pH.

Most compounds were tested at concentrations ranging from 100 to 6.25 µM, while Z-VAD-FMK was tested from 50 to 0.005 µM. For the proteasomal inhibitors, the enzymatic assay corresponded well with the yeast assay, with calpeptin as the most prominent drug while the boronic acid-containing compounds (bortezomib, delanzomib, ixazomib) had weaker but clear dose-response signal.

In contrast, simeprevir and Z-VAD-FMK which were marginal hits in the yeast assay, had a much stronger effect on MPro activity *in vitro*, indicating an inefficient uptake in cells (Fig. 6; Table 1). Lastly, oprozomib showed a weak dose response (Fig. 6; Table 1) and none was seen for carfilzomib (Table 1). This is in line with the yeast assay for those compounds, where no difference was seen between the tester strain and the control strain not expressing MPro (Fig. 5), indicating off-target effects for these compounds.

### Molecular docking of hit compounds in the SARS-COV-2 main protease binding site

**Fig 7:**
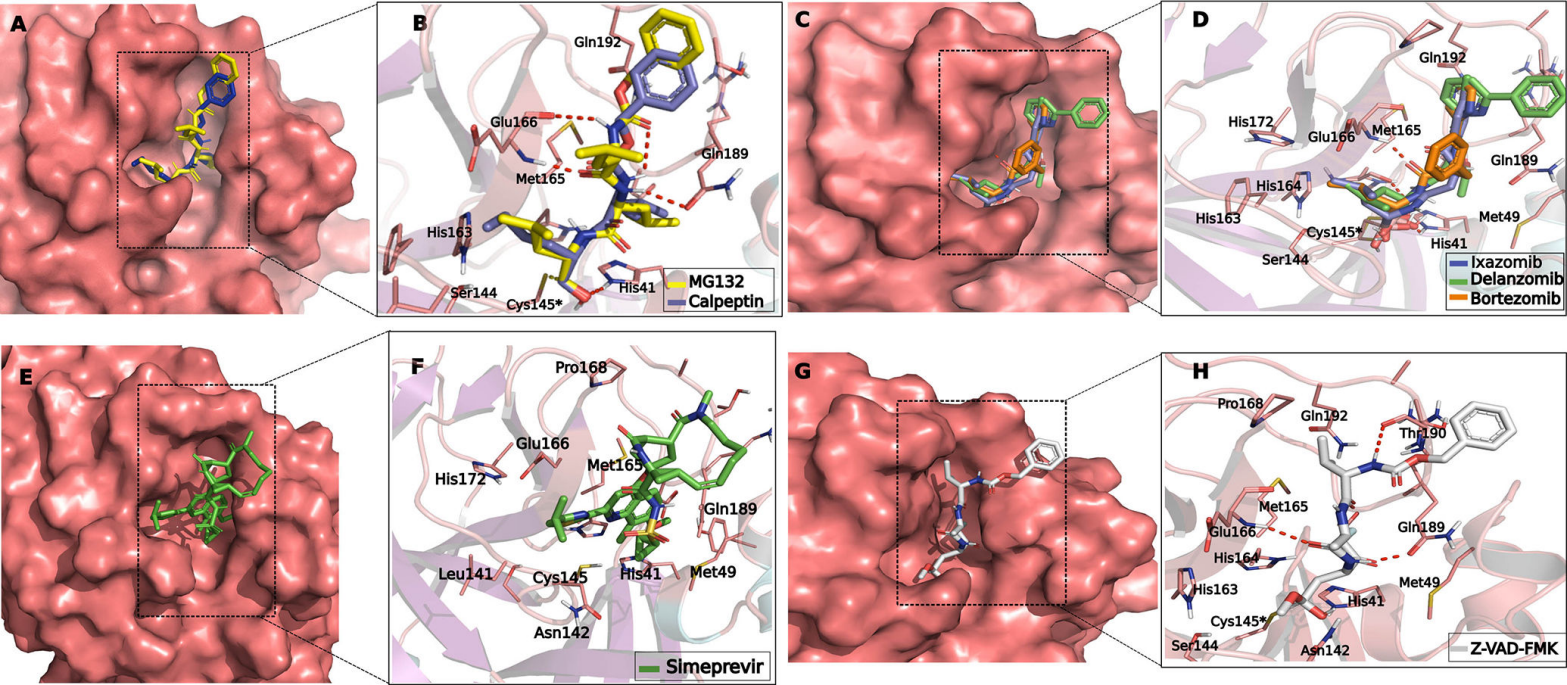
Docking of selected compounds giving an increase in growth in the yeast strain expressing MPro and the toxin chimera. (**A, C, E, G**) Compounds docked to the active site; (**B, D, F, H**) 2D diagram showing the same compounds with their respective interacting amino acids. (**A, B**) MG-132 (yellow) and calpeptin (blue). (**C, D**) Bortezomib (orange), delanzomib (green), and ixazomib (blue). (**E, F**) Simeprevir. (**G, H**) Z-VAD-FMK.

To investigate the interactions of the candidates obtained in the yeast-based assay (Fig. 3) to the MPro binding site (PDB ID: 7CB7) (36), molecular docking analyses were performed (Fig. 7), as described in Materials and Methods. First, we validated that the covalent docking method applied would be able to predict a covalent bond, performing the docking of the compound GC376 (aldehyde form), the original ligand of the protein structure 7CB7 (36), in our prepared protein model (Fig. S6A and B). The disposition of docked GC376 accurately predicts the pose of the same compound that was cocrystallized in complex with MPro (PDB ID: 7CB7) (36), as well as the covalent bond formed to Cys145 (Fig. S6A and B).

The MPro binding site has already been well described in previous studies (36, 37), and based on that we requested a covalent bond to be formed between the residue Cys145 of the protein and the reactive group of the compounds, if any. The nucleophilic addition reaction type was proposed for the interaction of our hits (Fig. 7), except for bortezomib, delanzomib, and ixazomib (Fig. 7C and D), where a boronic acid addition was proposed. The best-scored pose (Table 1) for each docked compound was selected for further evaluation of binding interactions. The results show that most of our ligands can smoothly fit in the MPro binding site (Fig. 7; Fig. S6). The molecular docking analysis suggested that the following compounds presented their reacting atoms within 5 Å from the S atom of Cys145 in the binding pocket of the SARS-COV-2 MPro, and are thus able to form a covalent bond (Table 1; Fig. 7): MG-132, calpeptin, bortezomib, delanzomib, ixazomib, Z-VAD-FMK, and oprozomib (Fig. S6C). Based on the same criteria, simeprevir (Fig. 7E and F), was unable to covalently bind Cys145 within 5 Å and was predicted as a non-covalent ligand (Table 1).

According to the covalent docking, the nucleophilic Cys145 interacts with the aldehyde group of MG-132 and calpeptin (Fig. 7A and B). The co-crystal for the compound MG-132 covalently bound to MPro was previously deposited (PDB ID: 7NG3) (22) and the binding mode observed in our docking studies is in concordance with the interactions previously reported for this compound and other proteasome inhibitors targeting SARS-COV-2 MPro (37). Other predicted interactions are important for the stabilization of MG-132 and calpeptin in the active site (Fig. 7B), such as the hydrogen bonds with His41, Glu166, and Gln189, and the hydrophobic interactions with Glu166 and Gln189. These same residues seem to be involved in the binding of oprozomib to the MPro active site (Fig. S6C and D).

The covalent bond between compounds bortezomib, delanzomib, and ixazomib (Fig. 7C and D) possibly occurs between the Cys145 and the boronic acid moiety of those compounds. Despite this difference, most of the residues predicted to interact with MG-132 and calpeptin also interact with the boronic-acid-containing compounds.

Regarding the model generated with the ligand Z-VAD-FMK, the reactive fluoromethyl ketone portion of the ligand is positioned within 5 Å from the nucleophilic S group of Cys145 in MPro (Fig. 7G and H), in a similar way to the available crystal structure from this compound bound to MPro (PDB ID: 7CUT) (37). Z-VAD-FMK also interacts with MPro through hydrogen bonds with Glu166, Gln189, and Thr190.

Finally, the docked structures obtained for simeprevir suggest that the compound can fit into the MPro binding site (Fig. 7E) and form a hydrogen bond with Glu166 (Fig. 7F). Nonetheless, simeprevir presented a low docking score (Table 1) and part of the simeprevir structure seems to be solvent-exposed (Fig. 7E). The molecular models proposed here, based on our docking analysis, are in general agreement with the MPro inhibition results obtained in the yeast-based assay and with previously reported MPro co-crystal structures.

## DISCUSSION

MPro has been the object of many target-oriented searches for antivirals against SARS-CoV-2. To identify MPro inhibitors *in vivo*, cellular screening systems have been set up using different approaches in mammalian and yeast systems, as well as infection models. For instance, reporter-based assays have been established in human cell lines (38–40), as well as in-cell protease assays (41). Another approach is represented by virus-infected human cell lines with target-based secondary assays (42). A yeast-based system with positive selection for GFP fluorescence has been implemented (16). The toxicity of MPro itself has also been used as a basis for *in vivo* negative selection systems in yeast aiming to map deleterious mutations in the protease (16, 43). Other screens have been based entirely on *in vitro* enzymatic inhibition assays, followed by X-ray crystallography of one MPro binder (23) or by a live virus assay (8). Screening using thermostabilization of MPro *in vitro* yielded proteasome and caspase inhibitors as candidates (37). Alternatively, *in silico* screening has been performed, followed by *in vitro* secondary assays including target protein binding and enzymatic inhibition (6) or antiviral assays (44).

Screens based on enzymatic inhibition *in vitro*, cellular assays, and infection models, provide different windows on the appropriateness of antiviral drug candidates. This work is based on a cellular assay screening system utilizing positive genetic selection in yeast. This approach also has limitations: first, the uptake of drug-like molecules in yeast cells is often limiting. Furthermore, no virus infection system is available, and the cellular environment of a yeast cell is not identical to that of a mammalian cell. From three partially overlapping compound sets of approved drugs and drug-like molecules, we identified eight candidate MPro inhibitors, of which three are novel.

### Identified candidate inhibitors

Most MPro inhibitors identified in our yeast test system were previously recognized as proteasome inhibitors, primarily composed of peptide derivatives (33). MPro inhibition has previously been reported in enzymatic assays *in vitro* for the aldehyde compounds GC376 (7, 20, 45), MG-132 (22, 23), and calpeptin (24, 46), the fluoromethylketone compound Z-VAD-FMK (37, 47), and Simeprevir (28–30). However, while previously identified in *in silico* screens to dock to MPro (25–27), this work is the first experimental report of the boron-containing bortezomib, delanzomib, and ixazomib functioning as MPro inhibitors.

*Bortezomib* promotes apoptosis and induces the unfolded protein response, and has been approved as a drug against myelomas and lymphomas (48). Bortezomib was previously tested in an *in vitro* MPro enzymatic assay for MPro inhibition but scored negative (49). *Delanzomib* is a derivative of bortezomib (50) with similar proapoptotic and antiproliferative effects. It can overcome bortezomib resistance in myeloma models (51). *Ixazomib*, when used clinically, is administered as a prodrug, a citrate ester of boronic acid. Similar to delanzomib, it is not cross-resistant with bortezomib.

### Discrimination of true positives in the genetic selection system

Features of the marker gene selection and reporter expression will lead to specific effects that have to be discriminated against for each screen using our system. We used the methionine-repressible *MET3* promoter to drive the expression of MazEF. Consequently, methionine or any molecule that can be metabolized to methionine, such as cysteine or glutathione, will suppress MazEF expression, leading to increased growth (Fig. 2A). Substituting the *MET3* for the *GAL1* promoter abolishes this effect, as expected (Fig. 2A). The plasmid expressing MazEF carries *URA3* as the selectable metabolic marker. Hence, the addition of uracil relaxes selection for the plasmid, leading to an increased fraction of plasmid-less cells in the population, again with increased growth as a consequence (Fig. 2B). These issues can easily be side-stepped by substituting marker gene or promoter, if required.

### The special case of proteasome inhibitors

It is reasonable to assume that the currently identified MPro inhibitors that were originally characterized as proteasome inhibitors to some extent will inhibit both these targets *in vivo*. In our assay, such small molecules may block the degradation of the fluorescent marker (mCherry), as the degradation of GFP and its derivatives is proteasome-dependent (52). We can, however, distinguish between these possibilities. Proteasomal inhibition without MPro inhibition would result in an increased signal in the fluorescent channel, independently of MPro. By contrast, inhibition of MPro should confer an MPro-dependent signal increase. By comparing the outcomes in strains expressing MPro or not, we see this directly (Fig. 5). Thus, oprozomib and carfilzomib also increased the fluorescence signal in the control strain lacking MPro at low concentrations while the absorbance signal decreased (Fig. 5), and they were cytotoxic at high concentrations in the tester strain (Fig. 3G; Fig. S2). This indicates that yeast proteasomes are the main target for these compounds.

### Boron-containing MPro inhibitors are only active in an enzymatic assay under non-standard buffer conditions

A compound that is active *in vitro* but not in a cellular system may simply display poor bioavailability. However, the inverse situation with target-directed activity *in vivo* but not *in vitro* is more remarkable. The boron-containing bortezomib and its derivatives delanzomib and ixazomib, and the epoxyketone compounds oprozomib or carfilzomib, all failed to inhibit MPro *in vitro* using standard conditions. However, when we modified the reaction buffer by excluding the reducing agent DTT, the MPro inhibitory activity of all three boron-containing compounds became evident also in an *in vitro* enzyme inhibition assay (Table 1).

Boronic acid molecules are in clinical use as anticancer agents, indicating that any side activities they might possess do not cause excessive toxicity. Boron compounds have also been tried as anti-infectives (53). Another structural class of boron-containing compounds, oxadiazaborole derivatives, show promise as MPro inhibitors according to *in silico* docking studies (54). Boronic acid exhibits selective bonding with diols, forming boronic or boronate esters. This property finds applications in sensors for biomolecule detection, including saccharides like glucose (55). Moreover, it serves as a component in drug delivery systems (56) and self-healing materials (57). Being both a thiol-containing reducing agent and a diol, DTT can interact with boronic acids through its thiol (–SH) and/or hydroxyl (–OH) groups. We speculate that this is why the boron-containing proteasome inhibitors fail to inhibit MPro when the buffer contains DTT. The structures of MPro co-crystallized with MG-132 in the presence (PDB 7BE7) compared to the absence (PDB 7GBP) of DTT (22) reveal only minimal differences, reinforcing the notion that direct inactivation of the boron-containing drugs by DTT is more likely to cause the inactivity of these compounds than changes in protein conformation.

It is noteworthy that this is not without precedent. In a study from 2004, it was found that bifunctional aryl boronic acid compounds were effective inhibitors of the SARS virus (SARS-CoV-1) MPro in an enzymatic *in vitro* assay (58). In that work, tris(2-carboxyethyl)phosphine, which is not a diol and contains no sulfhydryl group, was used as a reducing agent. Although not direct evidence, this is an indication that it may be the diol in DTT, rather than its reducing activity *per se*, that interferes with the action of boronic acid-containing MPro inhibitors.

Molecular docking studies have identified both bortezomib (3, 25) and ixazomib (27) as potential MPro inhibitors. Boronic acids can bind to nucleophilic residues, such as cysteine, and in particular serines (59). These boron-containing compounds form a reversible covalent bond to a threonine residue of chymotrypsin-like proteasome subunits (60). In the active site of MPro, there are three serines at positions 139, 144, and 147 (Fig. 7). However, the precise reaction mechanism for the inhibition of MPro or other cysteine proteases by boronic acid-containing molecules remains unknown. The prevailing model has the boron atom forming a reversible covalent bond with the sulfhydryl group of the protease catalytic triad, leading to a tetrahedral intermediate (61); this mechanism will have to be adapted according to the specific structure of MPro.

It is interesting to note that despite this *in silico* evidence, we have not found peer-reviewed publications describing anti-MPro activity *in vitro* for the boronic acid-containing drugs. Bortezomib was included in a series of compounds tested for MPro inhibitor activity in an enzymatic FRET-based assay and was reported as inactive (49). A screen of 5,000 pharmaceutical compounds for MPro inhibitors using an enzymatic assay found three fluoromethylketones including Z-VAD-FMK and a calpain inhibitor. However, none of the boronic acid-containing proteasome inhibitors were detected although bortezomib and ixazomib were represented in the library (47).

Together, these observations argue that while two classes of MPro inhibitors, aldehydes, and fluoromethylketones, work well in enzymatic inhibition assays *in vitro*, others, such as boronic acids, have problems functioning in the standardized buffer compositions used for many *in vitro* MPro enzymatic assays. We have changed the buffer composition and thereby found good *in vitro* conditions for the boron-containing drugs, and for those, the yeast assay results indicate MPro as the main target.

### Conclusions

We show here that the target-based cellular system can be used in high-throughput format for robotic screening of several thousand small molecules, and confers several benefits. Coupling protease activity to release of the powerful MazF toxin enhances sensitivity to MPro inhibition, and the yeast strain genetic background has been modified for increased uptake of external small molecules. Only bioavailable molecules will score in a cellular system, and the positive selection permits efficient discrimination between protease inhibitors and non-specific cytotoxic molecules.

Our system in yeast detected a number of previously identified MPro inhibitors. We also found three novel MPro inhibitors—boron-containing compounds that were previously selected by *in silico* screens but never scored in screens based on an *in vitro* enzymatic assay, using a standard buffer for all compounds to be tested. Only by eliminating DTT from the reaction could we demonstrate that this class of compounds is active as MPro inhibitors in an enzymatic assay. Combined, these advantages of this target-based cellular system give opportunities to detect some MPro inhibitors that are less efficiently detected using other systems. For viruses emerging in the future, our inhibitor screening system can be adapted for novel proteases to be targeted.

## MATERIALS AND METHODS

### Yeast strains and culture conditions

As an *S. cerevisiae* reporter strain, we used HA_SC_Met17_Mpro_Red carrying plasmid PSMv4 (17). This is constructed from strain 1352Y13363, which is sensitized to external small molecules through the *snq2Δ*, *pdr1Δ*, and *pdr3Δ* gene deletions. The reporter strain expresses MPro from the constitutive *Pichia GAP* promoter in the chromosomal *PDR3* locus. It also expresses a MazEF fusion protein with an MPro cleavage site inserted into the peptide linker connecting the MazE and MazF moieties from the weak and methionine-repressible *MET3* promoter (62) in a pCM188 (63) backbone. The red fluorescence marker mCherry is expressed from the strong *TDH3* promoter (32). The strain was maintained in Synthetic Defined (SD) medium [0.19% yeast nitrogen base, 0.5% ammonium sulfate, 2% glucose, and 0.077% Complete Supplement Mixture (ForMedium)] without uracil (SD–ura) supplemented with 400 µM methionine to suppress the expression of the toxin.

### Small molecule libraries

The COVID Box with 160 molecules implicated to have antiviral effects against SARS-CoV-2 was a gift from the Medicines for Malaria Venture. The L1035 Discovery Probe protease inhibitor library with 825 molecules and the L1021 Discovery Probe FDA approved Drug library with 1,971 molecules were purchased from ApexBio. There was compound overlap between the three libraries which allowed us to investigate consistency and batch-to-batch variation. The total number of unique compounds between these three libraries was 2,478. The libraries were provided as 10 mM stock solutions in DMSO and stored at −80°C. Before use, all compounds were first diluted in 100% DMSO to a concentration of 2.4 mM, and then further diluted to 30 µM by adding 1 µL into 80 µL culture medium with cell density OD_600 nm_ = 0.02.

### Phenotypic analysis in robot Eve

Growth of yeast strain HA_SC_Met17_Mpro_Red carrying plasmid PSMv4 was performed as described previously (17), except that the methionine concentration in the experimental growth medium was 10 µM. Overnight cultures were maintained at an exponential growth phase with methionine at 400 µM to reduce the expression of the toxin. Before the start of experiments, the media was removed and the pellet resuspended in SD–ura containing methionine to a final OD_600 nm_ of 0.02. Within the automated workstation, the culture was aliquoted into a Greiner 384-well black plate with a clear bottom using the Thermo Combi multidrop, and chemical compound libraries were diluted and transferred to the assay plate using the Bravo Liquid Handling platform to a final concentration of 30 µM of each compound (final DMSO concentration 1.25%) and a final volume of 81 µL. Growth at 30°C was stationary except for circular agitation at 1,000 rpm for 10 s, followed by 10 s in the reverse direction every 20 min. Compounds were tested in four replicates for each compound, and 36 replicates of the solvent-only control in each 384-well plate. Cell growth was quantitated with a BMG Polarstar Omega plate reader using 580 nm excitation/612 nm emission for at least 30 h. Reading and incubation cycles were integrated with the Overlord automation system (17, 32).

The yeast strain HA_SC_Red carrying plasmid pCM188-MET3 was maintained as mentioned above but was quantitated through both absorbance and 580 nm excitation/612 nm emission.

### Statistical evaluation of growth data

Raw data from the robot Eve was merged using a custom Python script. Growth curves, yield extraction, and statistics were performed using R software. A model was fitted to the growth measurements assuming sigmoidal growth with an exponentially decreasing intrinsic growth rate according to the Gompertz model (64) and maximum yield was extracted within the first 30 h. Two-sample *t*-tests comparing each compound to the solvent control were done and *P*-values were adjusted using the Benjamini and Hochberg false discovery rate [*P*-adj = P_i_ × N/rank_i_] (65). To visually inspect hits, growth curves were made using average relative fluorescence unit (RFU) ± SD from the quadruplicates, including the solvent control in the same plot as reference. To inspect the distribution of the phenotypic response, a scatterplot was made with average RFU and adjusted *P*-value. The R and Python software packages are listed in Table S2.

### *In vitro* enzymatic assay

SARS-CoV-2 Untagged 3-CL protease (catalog # 100823) was purchased from BPS Bioscience, San Diego, CA, USA. The fluorogenic peptide substrate Ac-Abu-Tle-Leu-Gln-AFC (66) was purchased from Biosynth, Bratislava, Slovakia. The initial assay was performed with 45 ng protease, 30 µM substrate, 50 µM fixed compound concentration, and 1 mM DTT in a 25 µL reaction volume using a 384-black well plate with a clear bottom, with a buffer composed of 20 mM Tris pH 7.8, 150 mM NaCl, 1 mM EDTA, and 0.005% Triton X-100. These parameters were optimized based on previously found conditions (35) (Fig. S3 Initial optimization). After initial optimization for the assay, further optimizations were done for the boron-containing compounds, where the omission of DTT and Tris/HCl at pH = 7.4 was found to result in stronger inhibition of the enzyme (Fig. S5). Fluorescence was measured with a BMG Polarstar Omega plate reader using 360 nm excitation/460 nm emission every 5 min for 90 min. Dose-response analysis of compounds with inhibitory effect was also performed at 6.25, 12.5, 25, 50, and 100 µM, alternatively 0.005, 0.05, 0.5, 5, and 50 µM depending on inhibitory effect at 50 µM.

### Molecular docking

The protein structure of SARS-COV-2 main protease (MPro) in complex with the inhibitor GC376 [PDB ID: 7CB7 (36)] was prepared with the Protein Preparation Wizard from the Schrödinger Suite version 2021-2 (www.schrodinger.com) applying the default parameters. Water molecules and ligands were removed, and energy minimization was performed by applying the OPLS4 force field. The compound structures (Fig. 4) in SDF format were prepared with the LigPrep tool in Schrödinger, attributing ionization states to pH 7.0. First, we performed an extra precision Ligand docking (XP docking, Schrödinger Release 2021-2) (67), defining the coordinates for the amino acid residues from the GC376 binding site in the 7CB7 crystal structure (His41, Phe140, Gly143, Cys145, His163, His164, Glu166, and Gln189). Moreover, covalent docking was performed (Glide Covalent Docking, Schrödinger Release 2021-2) (68) to investigate the formation of a covalent bond between the MPro structure (7CB7) and the candidates obtained by the yeast-based assay (Fig. 4). The centroid of the nucleophilic thiol (SH) group of the catalytic Cys145 was defined as the reactive residue for the grid box. The ligands were determined to react with the Cys145 through nucleophilic addition reaction type, except for bortezomib, delanzomib, and ixazomib, for which the boronic acid addition reaction types were proposed. The covalent bond was generated when the reacting pair of atoms between the ligand and the receptor were within 5 Å according to the reaction specified. To calculate binding free energies of the docked poses using the MM-GBSA, the covalent complexes were post-processed (Prime MM-GBSA, Schrödinger Release 2021-2) using VSGB as a solvation model (69). In order to select the best covalent docked complexes generated, the poses with the largest negative values of covalent docking affinity were chosen, and the poses were manually analyzed regarding the orientation of the ligand in the MPro binding site. Finally, MMGBSA binding free energies (dGbind) values were used to estimate the affinities of the ligands to the receptor, prior to the formation of the covalent bond (70).

### Overall procedure

A brief summary of the workflow is given in Text S1.

## Data Availability

Data from screens in yeast are available on GitHub. Raw growth data from the screen using robot Eve: https://github.com/sunnivass/Robotic_screen/tree/main/data. Scripts used for processing growth data: https://github.com/sunnivass/Robotic_screen. All docked best poses using XP and Covalent docking are available as free downloads at Zenodo.org, https://doi.org/10.5281/zenodo.7712368.

## SUPPLEMENTAL MATERIAL

Supplemental material spectrum.01249-24-s0001.docx Tables S1 and S2; Fig. S1 to S6.

ASM does not own the copyrights to Supplemental Material that may be linked to, or accessed through, an article. The authors have granted ASM a non-exclusive, world-wide license to publish the Supplemental Material files. Please contact the corresponding author directly for reuse.
