# Supplementary material for: An automated positive selection screen in yeast provides support for boron-containing compounds as inhibitors of SARS-CoV-2 main protease
Source: Microbiol Spectr. 2024 Aug 20;12(10):e01249-24. doi: 10.1128/spectrum.01249-24 (PMC11448104; doi:10.1128/spectrum.01249-24)
Supplement: Supplemental material — Tables S1 and S2; Fig. S1 to S6. [file spectrum.01249-24-s0001.docx]

**Supporting Information**

**Supplementary Table S1**: Experimental variation of a subset of compounds in the Mpro - MazEF selection system in yeast

| **Compound** | **Library 1** | **Library 2** | **Library 3** |  |
| --- | --- | --- | --- | --- |
|  |  |  |  |  |
| Bortezomib | 1.49 ± 0.03 *** | 1.32 ± 0.01 *** | 1.43 ± 0.01 *** |  |
| Delanzomib | 1.44 ± 0.03 *** | 1.40 ± 0.02 *** | 1.32 ± 0.02 *** |  |
| MLN2238 (Ixazomib) |  | 1.41 ± 0.02 *** | 1.47 ± 0.03 *** |  |
| MLN9708 (Ixazomib-citrate) |  | 1.40 ± 0.02 *** | 1.49 ± 0.03 *** |  |
| Simeprevir | 1.10 ± 0.02 *** | 1.04 ± 0.03 | 1.03 ± 0.02 |  |
|  |  |  |  |  |
| Boceprevir | 1.01 ± 0.01 | 0.75 ± 0.02 *** | 0.94 ± 0.01 *** |  |
|  |  |  |  |  |
|  |  |  |  |  |

Library 1: COVID BOX

Library 2: FDA-approved

Library 3: Protease Inhibitors

**Supplementary Table S2**: Software packages used

Scripts that were used in the analysis are available at <https://github.com/sunnivass/Robotic_screen/tree/main>

R packages used for scatterplot (Fig1), growthcurves (Fig1 and Fig2), quantification of growthcurves and t-test:

lubridate_1.8.0

ggplot2_3.3.5

Rmisc_1.5

tidyr_1.2.0

growthrates_0.8.2

deSolve_1.31

lattice_0.20-41

plyr_1.8.7

dplyr_1.0.8

data.table_1.14.2

reshape_0.8.8

Requirements for script used to merge growth measurement output from the Opentron automated cycler:

conda v4.10.3

Python v3.8.5

pandas v.1.2.3

**Supplementary Figure S1:** Pilot of primary yeast screen using fluorescence and absorbance readout


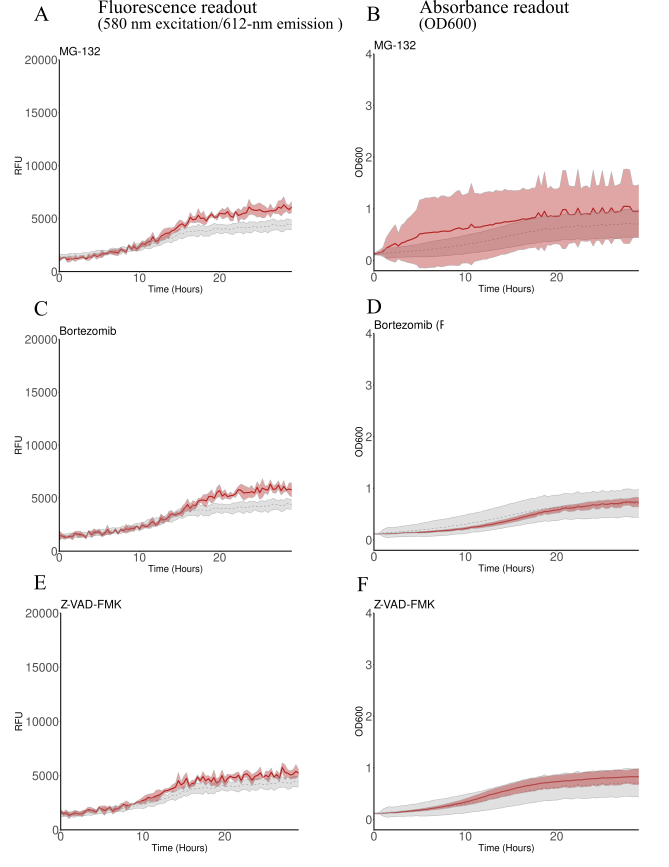


Growth curves for MG-132 (A, B), bortezomib (C, D) and Z-VAD-FMK (E, F) in the yeast reporter strain expressing MPro and the toxin chimera using fluorescent readout (A, C, E) or absorbance readout (B, D, F).

Grey curve with shaded area (standard deviation, SD) is growth of the control condition containing solvent only, red curve with shaded area (SD) is growth in presence of compound. For MG-132, the compound has a background signal in absorbance, masking the increase in growth. For bortezomib and Z-VAD-FMK, the absorbance signal is not different to the control condition, which has considerable variation.

**Supplementary Figure S2** Titration curves of carfilzomib in the yeast reporter strain expressing MPro and the toxin chimera.


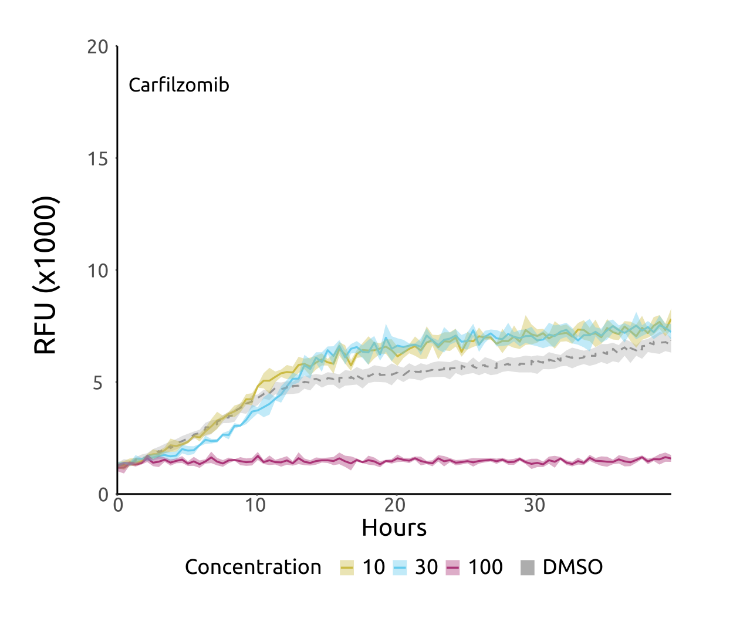


Grey curve is growth of the control condition containing solvent only. Both compounds show a small increase in growth at 10 (yellow) and 30 µM (blue) but are cytotoxic at 100 µM (pink).

**Supplementary Figure S3** Development of enzymatic assay


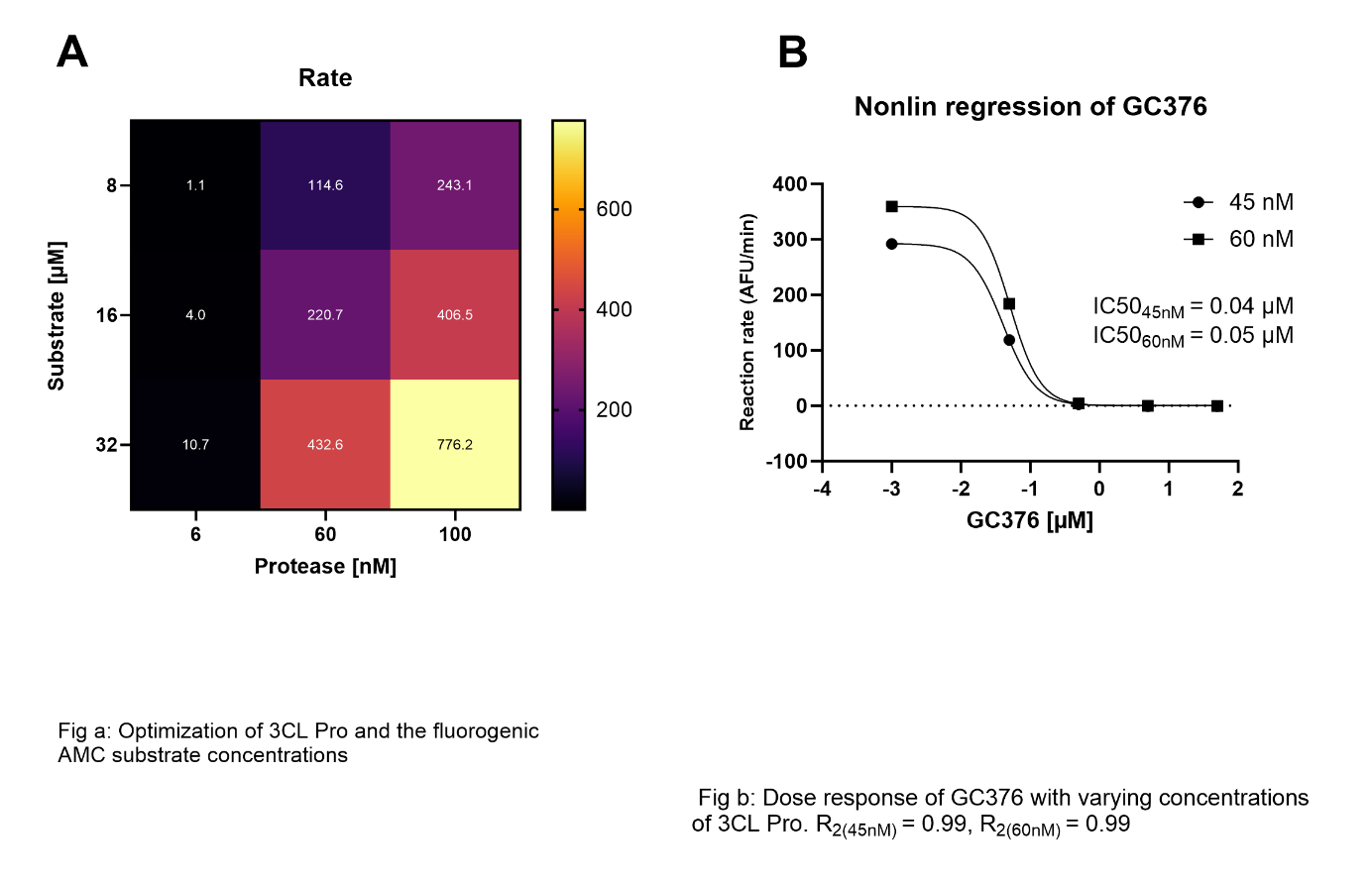


A) Concentration optimization of SARS-CoV 2 Untagged 3-CL protease (catalog # 100823, BPS Bioscience, San Diego, CA, USA) and the fluorogenic peptide substrate (Ac-Abu-Tle-Leu-Gln-AFC; Rut *et al.* 2020. bioRxiv, 2020.2003.2007.981928 ) for the enzymatic assay.

B) Dose response of GC376 with varying concentrations of SARS-CoV 2 Untagged 3-CL protease and the fluorogenic peptide substrate at 30 µM.

The fluorogenic peptide substrate (Ac-Abu-Tle-Leu-Gln-AFC; Rut *et al.* 2020. bioRxiv, 2020.2003.2007.981928) was previously found to be efficiently cleaved by the SARS-CoV 2 3-CL protease (Baker *et al.* (2021) PLoS One, 16, e0245962). To determine optimal concentrations of MPro and the substrate, we did a two‑dimensional titration (Supplementary Fig S5 A). We chose 60 nM MPro and 32 µM substrate to have a good activity with modest use of reagents. Next, we fixed the substrate concentration at 30 µM and performed dose-response curves of GC376 with 2 different MPro concentrations (Supplementary Fig S5 B). IC_50_ values at 45 and 60 nM protease were similar and therefore we chose to perform our assays at 45 nM protease and 30 µM substrate, again to reduce protease usage.

**Supplementary Figure S4** Biochemical activity of MPro in presence of candidate inhibitors and DTT


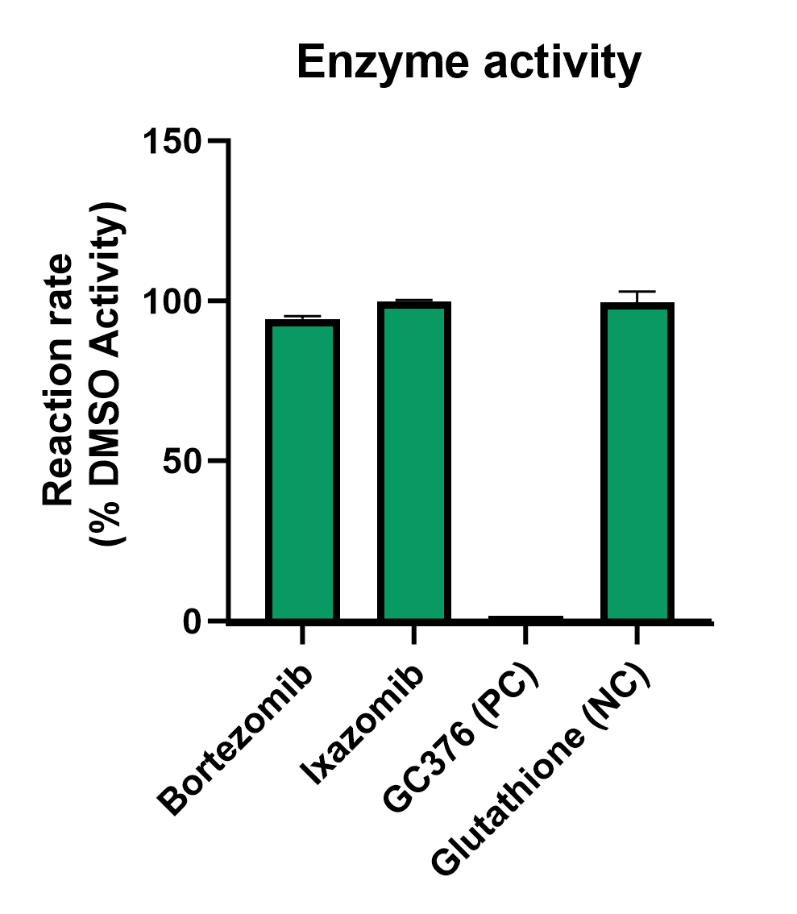


Reaction conditions: 50 µM inhibitor concentration, 30 µM substrate, 45 nM enzyme in standard assay conditions (20 mM Tris, 150 mM NaCl, 1 mM DTT, 1 mM EDTA, 0.005 % Triton X-100). Reaction volume 25 µl, kinetics measured every 5 min for 90 min using BMG Polarstar Omega plate reader at 360 nm excitation / 460 nm emission. Boxplot shows reaction rate (AFU/min) compared to samples with solvent only (DMSO).

**Supplementary Figure S5** Titration of DTT and pH conditions in the enzymatic assay


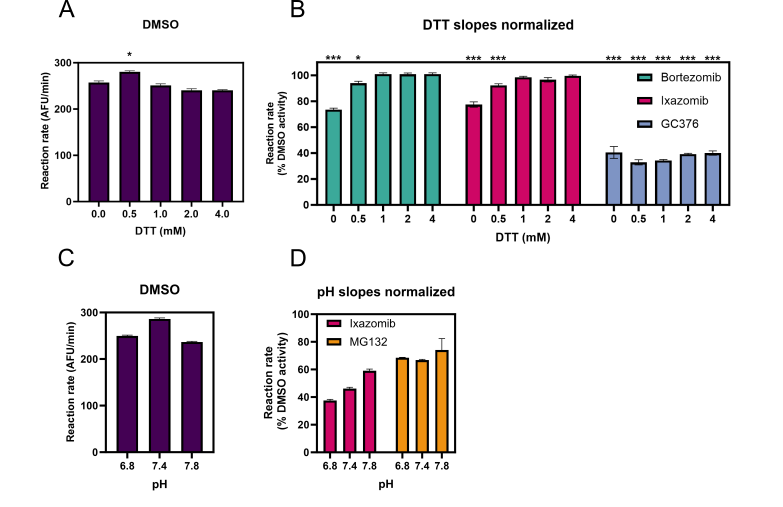


A) Activity of SARS-CoV-2 Untagged 3CL protease with varying concentrations of the reducing agent DTT in presence of the solvent only (triplicates). * the reaction rate at 0.5 mM was significantly higher than the others (Tukey’s 2way ANOVA P < 0.001)

B) Activity of SARS-CoV-2 Untagged 3CL protease with varying concentrations of reducing agent DTT in presence of candidate inhibitors Bortezomib (green, 50 µM), Ixazomib (pink, 50 µM) and GC376 (blue, 0.05 µM) (triplicates). Reaction rate is normalized to the control condition (DMSO solvent only). Asterices indicate significant difference to the control condition (Tukey's 2way ANOVA, *** P < 0.001, * P < 0.05).

C) Activity of SARS-Cov2 Untagged 3CL protease in the presence of the solvent only at varying pH levels (duplicates).

D) Activity of SARS-Cov2 Untagged 3CL protease with varying pH levels in presence of candidate inhibitors Ixazomib and MG132 at 50 µM (duplicates). Reaction rate is normalized to the control condition (DMSO solvent only).

Using standard assay condition with 1 mM DTT, candidate inhibitors bortezomib, ixazomib, and delanzomib had no effect (Supplementary Fig S4). Other studies have indicated that activity is dependent on the reducing agent (Kuzikov *et al.* 2021. *ACS Pharmacol Transl*, **4**, 1096-1110). We therefore titrated the DTT concentration at fixed compound concentrations. Enzyme activity did not differ with the exception of 0.5 mM DTT (Supplementary Figure S7 A, P < 0.001, Tukey’s 2way ANOVA). At high DTT concentrations, bortezomib and ixazomib did not inhibit MPro activity, while at 0.5 and 0 mM DTT they could effectively inhibit MPro activity (Supplementary Fig S7 B). We therefore chose to perform future assays without a reducing agent.

To investigate if the standard pH conditions affected MPro activity and inhibition, we titrated pH levels with fixed compound concentrations, using a known inhibitor (MG132) and a new candidate inhibitor (ixazomib). Here, activity of the control condition (DMSO only) was highest at pH 7.4 (Supplementary Fig. S7 C). MG132 inhibitory effect did not alter much, however the inhibitory effect of ixazomib increased with decreasing pH levels. Since enzyme activity was highest at pH 7.4, we decided to perform dose-response curves at this pH.

**Supplementary Figure S6** Docking of GC376, oprozomib, and carfilzomib


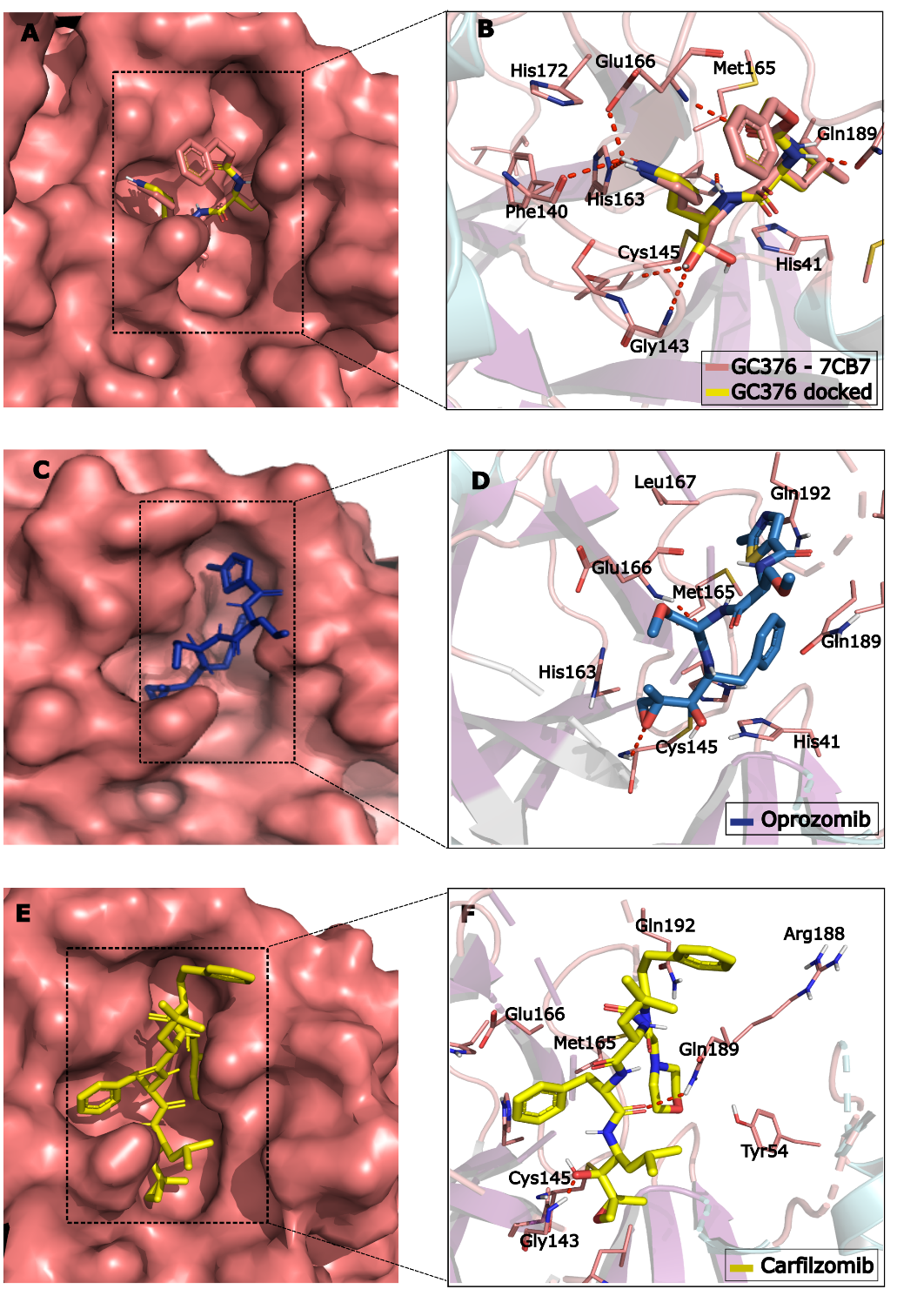


1. GC376 co-crystallized (orange) or docked (yellow) to the active site, B) same compound with interacting amino acids.

C,D) As A,B but for oprozomib

E,F) As A,B but for carfilzomib

**Supplementary text S1** Brief summary of the workflow in this paper

**I** Screening libraries of drugs and drug-like substances in genetically engineered *S. cerevisiae* strain. The strain expresses MPro, the red fluorescence protein mCherry, and also a chimeric protein consisting of the *E. coli* toxin MazF connected through a peptide linker with the cognate inhibitor MazE. In the linker, the recognition sequence for MPro is inserted. MPro cleavage in the linker releases MazF, inhibiting cell proliferation. External inhibitors reduce MPro cutting, promoting cell growth.

**II** This increase is monitored for the duration of a growth curve until stationary phase in a plate reader as higher fluorescence from mCherry. The curves were statistically evaluated and those showing significant increase were selected for follow-up tests.

**III** Initial hits were retested in yeast at different concentrations, and the fold change in growth yield was calculated.

**IV** Selected candidates from the retest in yeast underwent testing in an *in vitro* enzymatic inhibition assay with a fluorogenic substrate, spanning various inhibitor concentrations. Dose-response curves were plotted, and IC_20_ and IC_50_ values calculated.

**V** Promising inhibitor candidates underwent in silico dockings with substrate binding site of MPro.
